# Correlates of weapon carrying among high school students in the United States

**DOI:** 10.1186/1744-859X-7-8

**Published:** 2008-07-07

**Authors:** Adamson S Muula, Emmanuel Rudatsikira, Seter Siziya

**Affiliations:** 1 Department of Community Medicine and Public Health, University of Malawi, College of Medicine, Blantyre, Malawi; 2 Department of Global Health, School of Public Health, Loma Linda University, California, USA; 3 Department of Epidemiology and Biostatistics, School of Public Health, Loma Linda University, Loma Linda, California, USA; 4 Department of Community Medicine, University of Zambia, School of Medicine, Lusaka, Zambia

## Abstract

**Background:**

Deaths and injuries arising from interpersonal violence among adolescents are major public health concerns in the United States. The bearing of weapons among adolescents is a critical factor in many of these deaths and injuries.

**Methods:**

A secondary analysis of the 2005 United States Youth Risk Behavior Surveillance System Survey data was carried out to examine the variables associated with self-reported history of weapon carrying on school property among high school students. We used logistic regression analysis to assess the associations.

**Results:**

Of the 13,707 respondents who participated in the survey, 10.2% of males and 2.6% of females reported carrying a weapon on school property. In multivariate logistic regression analysis, males were more likely to report having carried a weapon than females (odds ratio (OR) = 5.58; 95% confidence interval (CI) [4.23, 7.62]). Self-reported race/ethnicity was also associated with weapon carrying. Other variables positively associated with weapon carrying at school were substance use (OR = 1.77; 95% CI [1.16, 2.68]), depression (OR = 1.44; 95% CI [1.10, 1.89]), suicidal ideation (OR = 1.64; 95% CI [1.23, 2.19]), having had property stolen or deliberately damaged at school (OR = 1.55; 95% CI [1.21, 1.98]), having been raped (OR = 1.70; 95% CI [1.22, 2.37]), having been threatened or injured with a weapon on school property (OR = 2.19; 95% CI [1.63, 2.95]), and having engaged in physical fighting (OR = 2.02; 95% CI [1.56, 2.63]).

**Conclusion:**

This research identifies factors that are associated with weapon bearing among adolescents in the United States. These factors may be important in the design of interventions aimed at improving school safety and adolescent health.

## Background

There is growing interest in the study of interpersonal violence among adolescents [1-3]. The carrying of weapons on school property by adolescents is an important aspect of adolescent physical and mental health. Adolescents carrying weapons on school property may cause physical injury and deaths among students and school staff [4]. Victims and witnesses of weapon use may also suffer psychological trauma from experiencing incidents where such weapons are displayed or used [5]. The learning environment within schools is disturbed if there is confirmed or suspected carrying of weapons by students.

In the recent past, there have been several high profile incidents in the United States that have highlighted the public health concern of weapon carrying among students. In 2004, the Centers for Disease Control and Prevention (CDC) estimated that 6.1% of high school students in the United States reported carrying weapons to school [6].

Previous research has reported that several factors are associated with carrying of weapons into schools. These factors include neighborhood and community factors, such as poverty and crime, family characteristics, school organization and climate. Individual level attributes such as gender, age and mental and psychological status of the person involved have also been reported to be associated with weapon bearing on school property [7-9].

In a study reported by Rudatsikira *et al*. [10] in which seventh to eighth grade students in school districts of the Southern California cities of Redlands and San Bernardino in the United States participated, 13.8% of the study participants reported having carried a weapon in the past 30 days. These authors also reported that previous victimization by the subject was associated with weapon carrying. A CDC report based on the 2005 Youth Risk Behavior Surveillance System (YRBSS) stated that "Overall, the prevalence of having carried a weapon was higher among male (29.8%) than female (7.1%) students" [11]. However, this CDC report did not assess whether being a victim of rape, having suffered deliberate property damage, having been threatened or injured and substance use were associated with carrying a weapon by the adolescent.

Based on the 2005 United States YRBSS, we carried out this study to report the prevalence of self-reported weapon carrying by in-school adolescents and to assess the association between weapon carrying and a selected list of explanatory variables such as race/ethnicity, sex, substance use, and previous victimization. The findings from this study may assist in the informed design of intervention programs aimed to reduce adolescent perpetrated injury and death resulting from weapon use.

## Methods

### Sampling strategy in the Youth Risk Behavior Surveillance System

This study was based on secondary analysis of data from the 2005 United States YRBSS. The YRBSS is an epidemiological surveillance system established by the CDC to monitor the prevalence of youth behaviors that most influence health [12]. The survey uses a three-stage cluster sample design to produce a representative sample of 9th through 12th grade students. In the 2005 survey, the target population consisted of all public, Catholic, and private school students in grades 9–12. Two hundred and three schools were sampled. In the first-stage, the sampling frame consisted of 1,261 primary sampling units (PSUs), consisting of counties, subareas of large counties, or groups of smaller, adjacent counties.

The 1,261 PSUs were categorized into 16 strata according to their metropolitan statistical area (MSA) status (that is, urbanicity) and the percentages of minority race/ethnicity groups (black and Hispanic students) in the PSUs. From the 1,261 PSUs, 57 were selected with probability proportional to overall school enrollment size for the PSU. In the second stage of sampling, 203 schools with any of grades 9–12 were selected with probability proportional to school enrollment size. The third stage of sampling consisted of randomly selecting, in each chosen school and in each of grades 9–12, one or two classrooms from either a required subject (for example, English or social studies) or a required period (for example, second period).

A weighting factor was applied to each student record to adjust for non-response and the oversampling of black and Hispanic students in the sample. The final, overall weights were scaled so that the weighted count of students was equal to the total sample size, and the weighted proportions of students in each grade matched population projections for each survey year.

In the 2005 survey, all regular public, Catholic, and other private school students in grades 9 through 12 in the 50 states and District of Columbia were included in the sampling frame. Puerto Rico, the trust territories, and the Virgin Islands were excluded from the sampling frame. Schools were selected systematically with probability proportional to enrollment in grades 9 through 12 using a random start.

All classes in a required subject or all classes meeting during a particular period of the day, depending on the school, were included in the sampling frame. Systematic equal probability sampling with a random start was used to select classes from each school that participated in the survey. Schools' response rate was 78% while students' response rate was 86%. The overall response rate was 67%.

### Questionnaire administration

Survey procedures for the national, state, and local surveys were designed to protect students' privacy by allowing for anonymous and voluntary participation. Before survey administration, the CDC Institutional Review Board approved the study protocol and parental permission was obtained by participating schools. Students completed the self-administered questionnaire during one class period and recorded their responses directly on a computer-scannable booklet or answer sheet. The core questionnaire contained 87 questions. States and cities could add or delete questions from the core questionnaire. Skip patterns were not included in any YRBSS questionnaire to protect student privacy by ensuring all students took about the same amount of time to complete the survey.

### Measures

A full presentation of the questions asked in the 2005 YRBSS has been reported by the CDC [12]. For the purpose of the current analysis, however, some of the questions of interest were as follow. With regards to weapon carrying, "During the past 30 days, on how many days did you carry a weapon such as a gun, knife, or club?" With regards to weapon carrying at school, "During the past 30 days, on how many days did you carry a weapon such as a gun, knife, or club on school property?" "During the past 12 months, how many times has someone threatened or injured you with a weapon such as a gun, knife, or club on school property?" "During the past 12 months, how many times has someone stolen or deliberately damaged your property such as your car, clothing, or books on school property?" "Have you ever been physically forced to have sexual intercourse when you did not want to?" A participant who reported "yes" to this last question was deemed to have been raped within the reported time period. The responses to these questions were dichotomized to yes or no; yes if the study participant reported experiencing an incident on at least one day and no if otherwise.

### Statistical analysis

We obtained frequencies of key variables in order to describe the study sample. Logistic regression analysis was conducted to determine associations between weapon carrying on school property and a selected list of potential explanatory variables. These variables, identified from previous research on adolescent violence, included: substance use [13]; depression [14]; suicidal ideation [15]; physical fighting [16]; and prior victimization, such as having had property stolen or deliberately damaged at school, having been raped, and having been threatened or injured with a weapon on school property [17-23]. Bivariate logistic regression analyses were conducted between each of the explanatory variables and the outcome (weapon carrying). Further, multiple logistic regression analysis was conducted with each of the explanatory variables as the main exposure while the other variables were considered potential confounders. Data were analyzed and weighted using the SUDAAN statistical software [24]. SUDAAN Programming statements for Taylor Linearization (DESIGN = WR) allowed for accommodation of the complex survey design [25] so that the results are representative of students in grades 9–12 in public and private schools in the US.

## Results

### Characteristics of the study participants

Data for 13,707 students who participated in the study were analyzed; of these participants, 6,664 (50.5%) were males and 7,193 (49.5%) were females (Table 1). The median age was 16 years (Q1 = 15 years, Q3 = 17 years).

**Table 1:** Characteristics of adolescents in the 2005 Youth Risk Behavior Surveillance System (United States)

Characteristic	Total % (n)	Males % (n)	Females % (n)
Age (years)			
<15	10.7 (1,221)	9.6 (520)	11.9 (701)
15	26.4 (3,170)	25.6 (1,450)	27.6 (1,720)
16	25.9 (3,535)	26.9 (1,718)	24.9 (1,817)
17	23.3 (3,661)	23.0 (1,780)	23.7 (1,881)
18+	13.6 (2,270)	14.9 (1,196)	12.3 (1,074)
Ethnicity			
White	67.8 (6,117)	68.3 (3,015)	67.4 (3,102)
American Indian or Alaska native	1.1 (144)	1.1 (86)	1.2 (58)
Asian	3.7 (366)	4.0 (187)	3.4 (179)
Black	16.0 (3,343)	15.6 (1,575)	16.5 (1,768)
Native Hawaiian or other Pacific Islander	0.9 (90)	0.8 (44)	1.1 (46)
Hispanic	10.5 (2,063)	10.3 (920)	10.6 (1,143)
Substance use (tobacco, alcohol, and illegal drugs)	85.9 (6,969)	86.7 (3,541)	85.1 (3,428)
Depression	28.5 (4,136)	20.4 (1,477)	36.7 (2,719)
Suicidal ideation	16.9 (2,330)	12.0 (772)	21.8 (1,558)
Property stolen or deliberately damaged on school property	29.7 (4,132)	31.4 (2,105)	28.0 (2,027)
Rape	7.5 (1,046)	4.2 (308)	10.8 (738)
Threatened or injured with a weapon on school property	7.9 (1,083)	9.7 (649)	6.1 (434)
Physical fighting	35.8 (4,862)	43.4 (2,877)	28.2 (1,985)
Weapon carrying on school property	6.5 (815)	10.2 (625)	2.6 (190)

### Prevalence of weapon carrying on school property

Of the 13,707 participants, 10.2% of males and 2.6% of females reported carrying a weapon on school property. An estimated 29.8% (95% confidence interval (CI) [28.4, 31.2]) of males and 19.3% (95% CI [17.4, 21.3]) of females had carried weapons anywhere.

### Factors associated with carrying a weapon on school property: bivariate analysis

Table 2 indicates that, according to bivariate analysis, there was no significant age difference in weapon carrying at school. Compared to female respondents, males were more than four times more likely to carry weapons to school (odds ratio (OR) = 4.22; 95% CI [3.40, 5.23]). Compared to whites, native Hawaiians or other Pacific Islanders were more likely to carry weapons at school (OR = 2.82; 95% CI [1.27, 6.27]). Other variables positively associated with carrying weapons at school were substance use (OR = 2.35; 95% CI [1.65, 3.51]), depression (OR = 2.43; 95% CI [1.94, 3.03]), suicidal ideation (OR = 2.75; 95% CI [1.86, 4.05]), having property stolen or deliberately damaged by peers on school property (OR = 2.42; 95% CI [2.02, 2.90]), physical fighting (OR = 4.48; 95% CI [3.00, 6.70]), having been raped (OR = 3.51; 95% CI [2.51, 4.90]), and having been threatened or injured with a weapon on school property (OR = 5.43; 95% CI [4.38, 6.74]).

**Table 2:** Unadjusted associations between weapon carrying on school property and selected characteristics among US high school students, 2005

	OR [95% CI]		
Characteristic	Total	Males	Females

Age (years)			
<15	1.00	1.00	1.00
15	1.19 [0.83, 1.89]	1.08 [0.69, 1.68]	1.14 [0.54, 2.37]
16	1.31 [0.92, 1.87]	1.28 [0.84, 1.95]	1.18 [0.58, 2.40]
17	1.19 [0.83, 1.71]	1.20 [0.78, 1.85]	1.07 [0.52, 2.20]
18+	1.29 [0.88, 1.89]	1.30 [0.83, 2.04]	0.75 [0.32, 1.79]
Gender			
Female	1.00		
Male	4.22 [3.40, 5.23]		
Ethnicity			
White	1.00	1.00	1.00
American Indian or Alaska native	1.19 [0.66, 2.16]	1.47 [0.78, 2.78]	0.06 [0.01, 0.45]
Asian	0.44 [0.18, 1.07]	0.32 [0.10, 1.07]	0.98 [0.32, 2.98]
Black	0.83 [0.64, 1.06]	0.65 [0.48, 0.90]	1.64 [1.06, 2.55]
Native Hawaiian or other Pacific Islander	2.82 [1.27, 6.27]	4.43 [1.77, 11.07]	1.65 [0.22, 12.26]
Hispanic	1.08 [0.82, 1.42]	1.15 [0.84, 1.58]	0.85 [0.46, 1.56]
Substance use (tobacco, alcohol, and illegal drugs)			
No	1.00	1.00	
Yes	2.35 [1.65, 3.51]	2.40 [1.52, 3.78]	2.20 [1.17, 4.15]
Depression			
No	1.00	1.00	1.00
Yes	2.43 [1.94, 3.03]	1.90 [1.58, 2.28]	3.67 [2.43, 5.55]
Suicidal ideation			
No	1.00	1.00	1.00
Yes	2.75 [1.86, 4.05]	3.33 [2.60, 4.25]	2.29 [1.88, 2.79]
Property stolen or deliberately damaged on school property			
No	1.00	1.00	
Yes	2.42 [2.02, 2.90]	2.25 [1.82, 2.77]	2.78 [1.90, 4.06]
Physical fighting			
No	1.00	1.00	1.00
Yes	4.48 [3.00, 6.70]	4.03 [3.20, 5.06]	4.84 [3.97, 5.89]
Rape			
No	1.00	1.00	1.00
Yes	3.51 [2.51, 4.90]	4.05 [3.09, 5.32]	2.60 [2.13, 3.17]
Threatened or injured with a weapon on school property			
No	1.00	1.00	1.00
Yes	5.43 [4.38, 6.74]	4.83 [3.75, 6.22]	5.79 [3.68, 9.12]

### Multivariate analysis of factors associated with carrying a weapon on school property

Overall, the main findings from the multivariate analysis were unchanged for age, ethnicity, gender, substance use, depression, suicidal ideation, property stolen or deliberately damaged on school property, physical fighting, having been raped, and having been threatened or injured with a weapon on school property (Table 3).

**Table 3:** Multivariate analysis of the associations between weapon carrying on school property and selected characteristics among US high school students, 2005

Characteristic	OR [95% CI]
Age (years)	
<15	1.00
15	0.91 [0.56, 1.47]
16	1.21 [0.76, 1.94]
17	1.18 [0.73, 1.94]
18+	1.21 [0.73, 2.03]
Gender	
Female	1.00
Male	5.68 [4.23, 7.62]
Ethnicity	
White	1.00
American Indian or Alaska native	0.82 [0.42, 1.60]
Asian	0.99 [0.35, 2.84]
Black	0.81 [0.58, 1.13]
Native Hawaiian or other Pacific Islander	4.67 [1.66, 13.14]
Hispanic	1.13 [0.82, 1.57]
Substance use (tobacco, alcohol, and illegal drugs)	
No	1.00
Yes	1.77 [1.16, 2.68]
Depression	
No	1.00
Yes	1.44 [1.10, 1.89]
Suicidal ideation	
No	1.00
Yes	1.64 [1.23, 2.19]
Property stolen or deliberately damaged on school property	
No	1.00
Yes	1.55 [1.21, 1.98]
Rape	
No	1.00
Yes	1.70 [1.22, 2.37]
Threatened or injured with a weapon on school property	
No	1.00
Yes	2.19 [1.63, 2.95]
Fighting	
No	1.00
Yes	2.02 [1.56, 2.63]

## Discussion

In a survey of US youth in 2005, 6.5% reported carrying a weapon to school. More males (10.2%) compared to females (2.6%) carried a weapon in the 30 days preceding the survey. Another 7.9% reported having been threatened or injured with a weapon, 7.5% having been raped and 29.7% having ever had property deliberately damaged or stolen at school.

There were sex differences with regard to the prevalence and effect of depression, suicidal ideation, physical fighting, victimization rape and having been threatened or injured with a weapon on school property. Females were more than twice as likely (10.8%) to report having been forced into sexual intercourse than males (4.2%). More males, however, were more likely to have reported being threatened or injured with a weapon compared to females (9.7% versus 6.1%). Slightly more males than females (86.7% versus 85.1%) were likely to have used substances (alcohol, tobacco or illicit drugs).

In bivariate logistic regression analysis, being male, native Hawaiian or Pacific Islander (whites as referent), substance use, depression, suicidal ideation, having been victimized (property stolen or deliberately damaged, raped or threatened or injured with a weapon), and physical fighting were all associated with weapon carrying on school property. In multivariable analysis, the results regarding the association between any of the explanatory variables and weapon carrying at school remained virtually unchanged.

The present study confirms previous reports on the predictors of weapon carrying among adolescents [10,13-23]. In a study sample of 3,054 high school students in Massachusetts, DuRant *et al*. [17] found that physical fighting was positively associated with weapon carrying on school property. Simon *et al*. [14] conducted a study of 2,200 participants and reported that depression and drug use in the 9th grade were predictors of handgun carrying in 12th grade. Using data from more limited sample sizes, Rudatsikira *et al*. [10] and Sullivan *et al*. [20] reported a positive association between victimization and weapon carrying among adolescents. While some of the previous studies included much smaller sample sizes derived from specific local settings, our study benefited from a much larger national sample that could be perceived as representative of the US school-going adolescent population.

The sex differences where males were more likely to report carrying weapons to school than females possibly has multi-factorial influences. Bailey *et al*. [26] reported that the perception that other students are carrying guns may be a major factor in adolescent weapon bearing. It follows, therefore, that male students who bear weapons may only be doing so out of belief that their peers are also carrying weapons. Furthermore, numerous studies have reported that males may be more likely to be violent and engage in other unhealthy and antisocial behaviors, such as cigarette smoking, alcohol and other substance use, and physical fighting, than females [27-29]. This may reflect the acceptance and/or expectation of the 'macho' status that males are renowned for.

We have also demonstrated that study participants who had suffered forced sex or had been victimized in other ways were more likely to carry weapons compared to those who had not. Several studies have reported that perceptions of fear and safety concerns, having being threatened with a weapon and feelings of vulnerability are associated with weapon bearing, possibly with the motivation of self defense [30-33].

Native Hawaiian and other Pacific Islanders and Hispanics were more likely to have reported having carried a weapon on school grounds compared to whites. Blacks on the other hand were less likely to carry weapons on school grounds compared to whites. There was no difference between whites and Asians. What do these results mean?

Firstly, we need to state that we do not believe that there are any inherent genetic differences that determine race and that affect the way that adolescents behave. There has been what we consider a healthy debate as to whether racial/ethnic disparities in health are due to either innate genetic differences or the biological impact of present and past histories of racial discrimination and economic deprivation [34-39]. While this debate has sometimes been polarizing and emotional, there has not been enough evidence to substantiate the claim that race has genetic roots rather than being a social construct; however, even as a social construct, it has enormous potential to influence individual and societal behaviors and perceptions. We take the view, therefore, that racial categorization has facilitated the distribution of social and economic resources (housing, school districts, wealth, social networks) that may consequently influence adolescent behaviors and perceptions toward violent behavior [40-43]. The perceptions that 'racial health disparities' are all genetic in origin and that race exists because of genetic differences have been discussed and possibly discarded by Krieger [44] and Kaufman and Cooper [45,46]. We take a similar view in interpreting the results of the current study.

We would have expected that minorities such as blacks (African Americans), being largely disadvantaged in the United States, would be more likely to bear weapons. This reasoning comes from the literature, where the poor are likely to live in violent neighborhoods and may be more likely to feel unsafe and therefore carry weapons to school. Our findings are therefore unexpected in as far as we had hypothesized that black adolescents will be at more risk of carrying weapons. There are possible explanations for these findings. Firstly, data were based on self-report. It is possible that black adolescents would under-report their weapon carrying experiences. However, we do not have evidence to suggest that this is the case. Secondly, if the black adolescents who are more likely to be carrying weapons have dropped out of school, then it is possible that the weapon-carrying adolescents would be under-represented. Thirdly, if black students felt less threatened at school, they may not thus carry weapons. Fourthly, black students may not carry weapons to school if the schools they attend are more vigilant in policing weapon carrying as a possible result of high violence and weapon bearing in black neighborhoods; if we knew this to be the case, it would suggest that such an intervention was effective. Finally, we may have been biased into thinking that blacks are more likely to carry weapons as a result of prevailing prejudices. However, we also do not intend to minimize the significantly higher risk of violent death by firearms among blacks compared to whites in the United States [47-50], although, with regard to our study, these data are limited by being largely from situations outside of school and among adults. These studies also report solely firearm injuries and deaths while our study includes all weapons, not just firearms. The higher likelihood of carrying a weapon among Hispanics may have similar associations with poverty or social factors.

Using students less than 15 years old as referents, we found that older students were more likely to report having carried weapons on school grounds. This could mean several things: that older adolescents may be at higher risk of perpetrating violence or being victimized; that older adolescents may be more likely to resort to violence to resolve conflicts [51]; or that peer influence or acceptance of weapon bearing is higher in this age group. Furthermore, older adolescents may be more likely to be successful in concealing weapons than much younger students.

The fact that adolescents who reported having suicidal ideation and depressive symptoms and substance use were also likely to bear weapons may be an example of 'clustering of harmful health behaviors' [27,52-56]; that is, an adolescent who uses substances is also likely to bear arms. As a policy direction, this calls for adolescent health intervention program policy makers and implementers to seriously consider that an adolescent who has one particular unhealthy behavior may also have other behaviors that need attention. A multi-problem and multifaceted approach has a better chance of promoting health than single vertical interventions.

### Limitation of the study

Although the current analysis has strengths, there are also some limitations that need consideration. Firstly, the data used in the analysis were based on self-reports; therefore, bias could result from possible mis-reporting by participants, either intentionally or inadvertently. Furthermore, the response rate for this survey was 67% of all the eligible students. The experiences of the students who did not respond are not known. If, however, these non-responders had different experiences and exposures, our study may not be representative of the situation among US adolescents in schools. Finally, a small but not insignificant percentage of adolescents in the US are either home schooled, are truant or do not attend school altogether. Our findings may not be applicable to this group. It is also important to highlight the fact that data for this study were collected from a survey (cross-sectional methods) and so it is not possible to attribute causation to any of the factors so far identified as associated with weapon carrying [57-60].

## Conclusion

This research identifies factors that may be used in the design of interventions aimed at improving school safety. Knowledge that males are more likely to carry weapons may enable the targeting of boys in prevention programs. Substance use prevention among adolescents may also be associated with reduced weapon carrying. We believe the racial/ethnic differences may be indicative of general socio-economic disparities existing across neighborhoods in the US. The mechanisms behind the disparities in weapon carrying with regard to socio-demographic characteristics also need to be explored.

## Competing interests

The authors declare that they have no competing interests.

## Authors' contributions

ASM participated in the interpretation of the results and drafting of the manuscript, ER conceived the analysis plan, conducted the statistical analyses and participated in the drafting of the manuscript, SS participated in the interpretation of the results and drafting of the manuscript. All authors approved the manuscript.
